# The Australian Community Does Not Support Gender Selection by IVF for Social Reasons

**DOI:** 10.1155/2013/242174

**Published:** 2013-12-17

**Authors:** Kovacs Gab, McCrann Julian, Levine Michele, Morgan Gary

**Affiliations:** ^1^Monash IVF, Epworth Hospital, Bridge Rood, Richmond, VIC 3121, Australia; ^2^Roy Morgan Research, 401 Collins Street, Melbourne, VIC 3000, Australia

## Abstract

This study was carried out to determine the attitudes of the Australian community to IVF by a reliable community poll. Cross-sectional surveys, conducted by telephone of a random sample of 650 Australians were undertaken. The sample was drawn from the residential phone numbers in the Australian electronic “White Pages” and stratified by geographical area with quotas controlled by gender and age to be representative of the Australian population. The participants were asked to answer to three questions about gender selection, and their response was measured as “yes-allowed,” “no-not allowed,” or “undecided” for each of the questions. Whilst 91% of respondents supported the use of IVF to help infertile couples, only 20% supported gender selection within IVF or for family balancing. When it came to the use of IVF only for gender selection, only 17% were in favour. This survey shows that Australian community overwhelmingly opposes gender selection for social reasons.

## 1. Introduction

With the development of preimplantation genetic diagnosis (PGD) using IVF technology, it is now technically possible to determine the gender of the embryo before being transfered with a high degree of certainty [1]. There has been much discussion in the media whether the technique should be allowed. In the State of Victoria gender selection is only permitted by legislation for “medical indications” [2], and throughout Australia it is against the guidelines of the National Health and Medical Research Council [3], thus preventing Australian couples from using this technology. Whilst the technique is forbidden in India and the European Community, it is permitted in several countries. The ethics of clinicians referring couples for gender selection from countries where it is forbidden to other countries where it is permitted and is performed has recently been the subject of debate [4].

Opinion of Ethics Committees can reflect the attitudes of its membership, or they can be swayed by vocal minorities. We carried out a survey by an experienced and reliable “Gallup Poll” organization to assess the attitude of the general Australian community on social gender selection.

## 2. Methods

As part of the regular Morgan Gallup polls three questions with respect to IVF and gender selection were included with the Morgan Gallup Telephone Poll of the week of February 1st, 2011.

Morgan Gallup Polls are carried out each fortnight as cross-sectional surveys, conducted by telephone of a random sample of 650 Australians. This survey was conducted as part of a larger omnibus community survey performed by Roy Morgan Market Research (Melbourne, Australia) about voting intentions and consumer preferences. The sample was drawn from the residential phone numbers in the Australian electronic “White Pages” and stratified by geographical area with quotas controlled by gender and age to be representative of the Australian population. Multiple attempts were made to contact each phone number that was randomly drawn at different times on different days.

The survey was carried out by asking three questions to determine the respondents' attitudes to gender selection; the questions in order are presented below, with an Australia wide cross section of 650 respondents aged 14 years and over.

These were as follows.

1. “It is now possible for people having IVF treatment to decide the baby's sex (gender selection). At present gender selection is not allowed. In your opinion should people having IVF treatment be allowed to select the gender of their baby or not?”
2. “Couples who are not infertile can determine the sex of their baby using IVF (gender selection). In your opinion, should gender selection be allowed for anyone?”
3. “Should couples who already have one or more child of one sex be allowed to use the gender selection technology to select the gender of their next child (family balancing)?”

The attitudes of respondents were measured as “yes-allowed,” “no-not allowed,” or “undecided” for each of the questions. Analysis of responses by several variables was carried out.

As this study contains no identifying material, and it conforms to the standards established by the NHMRC for ethical quality, ethics approval was not sought.

## 3. Results

The overall results for the three questions are summarized in Table 1.

The responses were analyzed by age and gender in Table 2.

The responses were compared between residents in capital cities and rural inhabitants and shown in Table 3.

The responses were also analyzed by educational status. However, the numbers analyzed here are small and at best are indicative but certainly not significant. Interestingly, respondents who only had a primary school education (101 respondents) were more liberal and had a significantly higher rate of “allowed” responses (35.4% for question 1, 31% for questions 2 and 3), whereas current university students (31 respondents) were much more conservative (12.8% “YES” for question).

## 4. Discussion 

It appears from our study that the majority of Australians in 2011 agree with the members of the Health Ethics Committee that social gender selection should not be permitted, as 73% of all respondents responded that it should “not be allowed.”

The prohibition of gender selection in Australia was recommended by the Health Ethics Committee of NHMRC in 2004. Why did the NHMRC prohibit gender social selection? In appendix 1 of the document (NHMRC) there are three reasons given.

The first was that parental love should be unconditional acceptance and not depend on the sex of the child—“Sex selection is incompatible with the parent-child relationship being one that involves unconditional acceptance.”

The second was, that sex selection may be an expression of “sexual prejudice, in particular against girls.” If male children are chosen in preference, this denigrates the value of females. “As practiced today around the world, it generally reflects and contributes to bias and discrimination against women.”

The third was, that the natural sex ratios may become unbalanced if there is preference for a particular sex selected by this technique. “Sex selection harms men in some cultural groups (by contributing to the shortage of women for men to marry).”

Are these reasons valid and do they mirror the opinions of the community?

“Unconditional love” is an excellent “motherhood statement” in theory, but does it always apply in practice? May there be some situations when the community would condone gender selection, such as where a couple has several children of one sex already (family balancing) or where they have lost a child of one sex which they would hope to “replace”?

Secondly, in some countries there is a preference for boys, but this is not the case in Australia, where requests for female children are just as common. Therefore the denigration of females does not apply.

Thirdly, although 1 in 30 children in Australia is now conceived by IVF, a very small percentage would choose gender selection if available, and the numbers would be too small for “unbalancing the sex ratio” to happen.

The reasons given for permitting gender selection are that it “may enable parents to fulfill religious obligations or cultural expectations,” and that selection of gender is properly thought of as a matter for individual autonomy.

Interestingly, our 2011 survey is in exact agreement with an Australian mail-out survey of social attitudes carried out back in 2006, but published recently, [5] where it was found that 69% of respondents disapproved or strongly disapproved of the use of IVF for sex selection. It would appear that despite repeated discussion in the media, as detected by this survey, the community's attitudes have not changed during the last five years with the overwhelming opinion in the Australian community being against gender selection. Whether this is due to a lack of informed discussion or because of its association of eugenics using “the thin edge of the wedge” principle is unknown. Liberal ethicists, who are proponents for gender selection and maintain that the technique should not be forbidden as it does not harm anybody, have not been able to get their message accepted by the community.

It is noted that amongst younger respondents (18–34 years) up to 31% supported one or other of the options, which may mean that attitudes may change in the future. However, this is only speculating and it is clearly is not possible to measure accurately what people's opinions may be five or ten years hence!

## Figures and Tables

**Table 1:** The attitudes of respondents overall.

	Allowed (%)	Not allowed (%)	Undecided (%)
Question 1	20.7	73.4	5.9
Question 2	17.6	76.5	6.0
Question 3	20.2	74.2	5.6

**Table:** (a) Question  1

	Men	Women	14–17	18–24	25–34	35–49	50–64	Over 65
Sample size	300	350	46	60	88	174	168	114
Approve	22.2	19.3	27.7	30.6	27.8	18.0	15.7	18.6
Disapprove	73.9	72.8	72.3	64.1	62.8	78.4	75.0	77.3
Undecided	3.9	7.9		5.3	9.4	3.7	9.3	4.2

**Table:** (b) Question  2

	Men	Women	14–17	18–24	25–34	35–49	50–64	Over 65
Sample size	300	350	46	60	88	174	168	114
Approve	20.3	14.9	28.3	30.8	27.2	14.2	11.3	12.8
Disapprove	74.7	78.1	71.7	62.2	69.4	82.0	80.2	77.2
Undecided	5.0	6.9		7.0	3.4	3.8	8.6	10.0

**Table:** (c) Question  3

	Men	Women	14–17	18–24	25–34	35–49	50–64	Over 65
Sample size	300	350	46	60	88	174	168	114
Approve	22.6	18.0	27.6	30.1	31.3	16.6	14.4	17.1
Disapprove	71.1	77.2	69.7	62.6	65.0	77.6	80.5	75.3
Undecided	6.3	4.8	2.7	7.2	3.7	5.8	5.1	7.7

**Table 3:** Comparison of city and rural residents' responses.

	Capital city respondents (419) (%)	Rural respondents (231) (%)
Question 1		
Allowed	22.4	17.9
Not allowed	71.0	77.2
Undecided	6.5	4.9

Question 2		
Allowed	18.0	16.9
Not allowed	76.0	77.3
Undecided	6.0	5.8

Question 3		
Allowed	20.3	20.1
Not allowed	73.6	75.1
Undecided	6.0	4.9
